# Radial Distance Estimation with Tapered Whisker Sensors

**DOI:** 10.3390/s17071659

**Published:** 2017-07-19

**Authors:** Sejoon Ahn, DaeEun Kim

**Affiliations:** Biological Cybernetics Lab, School of Electrical and Electronic Engineering, Yonsei University, Shinchon, Seoul 120-749, Korea; ahn.sj@yonsei.ac.kr

**Keywords:** tactile sensor, artificial whisker, rat whisker, active sensing

## Abstract

Rats use their whiskers as tactile sensors to sense their environment. Active whisking, moving whiskers back and forth continuously, is one of prominent features observed in rodents. They can discriminate different textures or extract features of a nearby object such as size, shape and distance through active whisking. There have been studies to localize objects with artificial whiskers inspired by rat whiskers. The linear whisker model based on beam theory has been used to estimate the radial distance, that is, the distance between the base of the whisker and a target object. In this paper, we investigate deflection angle measurements instead of forces or moments, based on a linear tapered whisker model to see the role of tapered whiskers found in real animals. We analyze how accurately this model estimates the radial distance, and quantify the estimation errors and noise sensitivity. We also compare the linear model simulation and nonlinear numerical solutions. It is shown that the radial distance can be estimated using deflection angles at two different positions on the tapered whisker. We argue that the tapered whisker has an advantage of estimating the radial distance better, as compared to an untapered whisker, and active sensing allows that estimation without the whisker’s material property and thickness or the moment at base. In addition, we investigate the potential of passive sensing for tactile localization.

## 1. Introduction

Many animals have particular sensor systems to sense their environment. Bats are well known to use echolocation system with ultrasonic sensors [1]. Weakly electric fish living in turbid water generate electric discharge and read an image of electric field in order to catch their prey [2]. Sand scorpions can detect surface waves of vibration caused by prey movement [3]. Rodents are known to use their whiskers to recognize objects in the environment [4]. Harbor seals also use their whiskers to detect water trails generated by their prey fish. [5,6,7]. Rats actively move their whiskers while the harbor seal does not [8,9,10,11].

Rats use their whiskers for texture discrimination or feature extraction of nearby objects. Many researchers have attempted to apply the whisking property into biomimetic systems by designing artificial whisker sensors which could discriminate different textures [12,13], estimate radial distances [14,15,16,17,18,19], object features [20,21] and terrain mapping [22].

In the attempt to mimic the ability of the rat’s texture discrimination, Fend et al. [23] used a whisker sensor attached to a mobile robot in order to perform texture discrimination. Kim and Moeller [24] also performed a texture discrimination task using a biomimetic whisker sensor. Lepora et al. [12,13] attached a whisker sensor to a Roomba robot and swept the floor surface and classified different types of surfaces using the whisker deflection measurement. The classification was based on Naive Bayes algorithm.

One of the most popular applications of the whisker sensor is radial distance estimation, which estimates the Euclidean distance between the base of the whisker and the contact point with a target object. Birdwell et al. [17] showed a method to estimate radial distance using a tapered whisker deflection and a method using moment and angular velocity of the whisker. Novel methods for estimating the radial distance were also made using the tapered whisker [18]. While many methods for contact point estimation are based on the linearized Euler-Bernoulli equation, some methods are based on the nonlinear Euler-Bernoulli equation. Methods based on the nonlinear Bernoulli-Euler equation using torque and force measurements reconstructed the whisker shape, which allowed it to find the radial distance. One study was implemented to measure radial distances of an object in 2-D space [25], while another was for measuring radial distances of an object in 3-D space [15].

Also, the antenna of insects have been mimicked and a mathematical model was introduced to describe the dynamics of the antenna sensor when sensing or estimating radial distance, where the torque information is crucial [16,26,27,28]. Objects which have no sharp edges could often occur longitudinal slip or lateral slip. Analysis of slip was given in the work by [19]. Solomon and Hartmann proposed a method to estimate contact point location, even in situations where lateral slip exists when a good estimation of the friction coefficient could be made [14]. They also have made an algorithm which could account for longitudinal slip by constantly updating the contact positions using torque information [21]. In these works, torque information was crucial as well. Instead of using torque or force measurements, by measuring a deflection angle at a point of the whisker sensor, the radial distance could be estimated even without knowing the rotational stiffness of the whisker [19,24,29].

The whisker sensors have an advantage over other proximity sensors including laser, sonar and infrared sensors. They can be relatively easily designed with a cheap cost. They can provide a time course of distance information in a sweeping mode and furthermore shape information [19]. In a close distance, multiple whiskers can provide spatial information for the surrounding environment. The tactile signals are invariant to the environmental conditions. In contrast, infrared or sonar sensors are affected by what type of objects are probed, and their reflected signals may change.

Recently, there have been many studies to develop artificial whisker sensors, inspired by both rat whisker and seal whiskers [30,31,32]. Interestingly, the whisker sensors with an undulatory form to model harbor seal whiskers can reduce vortex-induced vibrations in the underwater [30].

Whisker sensors were also used in other applications, such as air flow measurements. Solomon and Hartmann [20] have proposed that a whisker sensor array, where the individual whisker shaft’s cross section is similar to a long rectangle, could accurately measure a stationary air flow velocity when it is blowing perpendicular to the sensor shaft. Rooney et al. have attached an actively moving whisker sensor on a moving underwater robot in order to guide the robot [33]. Schultz et al. [22] has proposed various types of applications which may be appropriate for a hovering robot in Mars, such as rover speed estimation, wheel slip detection, surface roughness measurement.

Many studies are also related with whisker morphology and the mechanical characteristics. Towal et al. [34] quantified the morphology of rats’ whiskers using 3D and 2D scans. They set up equations to approximate rat whiskers’ location and shape. Such approximation of whisker array morphology into a set of equations, and such models could simulate how the rats will sense objects. Also, the characteristics of the actual rat’s whiskers have been investigated. Hartmann et al. investigated the mechanical characteristics of whiskers including the resonant frequencies depending on each whisker [35] while Quist et al. have shown that Young’s modulus varies [36]. The rats are known for their ability to discriminate different types of textures [37,38,39].

**Figure 1:**
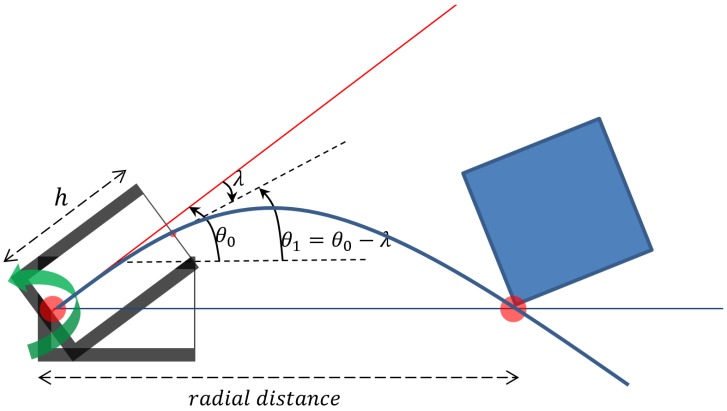
The schematic of the active whisker sensor system. Radial distance is defined as ’distance between whisker sensor’s pivot point to whisker-object contact point. $\theta_{0}$ denotes the protraction angle, λ is the deflection angle measured by sensors at position *h*, and the tangential angle at sensor $\theta_{1}$ is calculated as $\theta_{1}=\theta_{0}-\lambda$.

Figure 1 shows a schematic diagram for whisker contact with an object. Here, we assume that there is a single point contact with a target object since the whisker has bending stiffness. If the target object is round, the contact point on the whisker will shift during active whisking, and the contact distance from the base will change. It may provide the shape information of the target. If the target object has a sharp corner, the contact distance will be unchanged as the whisker slides over the object surface. Here, we assume that the whisker has a point contact for distance estimation. Furthermore, a set of whiskers can collect more information about a target object or the surrounding environment. Multiple whiskers are helpful to detect the distance of a target object and recognize its shape [19]. Interestingly, it is reported that there are parallel neural pathways for signaling multi-whisker signals to the barrel cortex [40,41].

Williams and Kramer [42] showed advantages of a tapered whisker. First, maximal deflection and protraction of a tapered whisker were compared with a cylindrical (untapered) whisker and it was concluded that tapered whiskers have a smaller maximum deflection angle, and a smaller maximum protraction angle which may increase the spacial acuity. Also, the rotational stiffness was compared with both types of whiskers. It was shown that the tapered whisker’s rotational stiffness changed more drastically compared to the cylindrical whisker. Finally, it was shown that tapered whiskers’ natural frequency varied less compared to the cylindrical whisker when both had a tip break.

In this paper, we handle tapered whiskers, which more closely resemble real animal whiskers, and derive an analytic model to estimate the radial distance. We first check the noise robustness of the analytical model which can estimate the radial distance under active sensing using two angle information, protraction angle and deflection angle. We take a SNR test on the tapered whisker model for noise sensitivity analysis. We apply the nonlinear Bernoulli-Euler equation for large deflection angles, since the linearized equation assumes that the deflection is small. To quantify the error of radial distance estimation, the analytical data will be compared with numerical nonlinear solutions even with large deflection angles.

Here, we argue that tapered whiskers have advantages in the sense that they are more robust to noise when estimating the radial distance. Noise robustness of the tapered whisker and the cylindrical whisker will be compared in simulation experiments. We note that active sensing can play a crucial role in estimating the radial distance. We also propose that by using two different deflections on the whisker, it is possible to estimate the distance. Advantages of the tapered whisker will be discussed in details by analyzing our results.

## 2. Methods

Active whisking is one of interesting properties observed in real rodents. To analyze the radial distance estimation of whiskers, we handle three types of whisker models. To see the deflection property of the whisker depending on the contact distance, we use Bernoulli-Euler equation.

### 2.1. Linear Cylinder-Type Whisker

Using the protraction angle and deflection angle at the location of the sensor on the whisker, the radial distance can be estimated [19,24,29]. This analytical model assumes that the whisker is under active sensing, which means it rotates the whisker for contact. The distance from the base of the whisker to the contact point of an object is called radial distance. The relationship with the tangential angle at an arbitrary location *x* of the whisker or radial distance can be expressed as

(1) $$EI\tan\;\theta =\frac{\tau }{2d}x^{2}-\tau x+\frac{1}{3}\tau d$$

where *E* is Young’s modulus, *I* is the moment of inertia, θ is the tangential angle at *x* and τ is the bending moment (rotational force) at whisker base.

We denote the protraction angle as $\theta_{0}$ and the tangential angle at sensor postion x=h as $\theta_{1}$ (where $\theta_{1}=\theta_{0}-\lambda$, and λ is deflection angle at a position *h*) and obtain the Equation (2).

(2) $$\frac{\tan\;\theta_{1}}{\tan\;\theta_{0}}=\left(\frac{3h^{2}}{2d^{2}}-\frac{3h}{d}+1\right)$$

We can choose another sensor position rather than the whisker base (x=0). Two sensor positions ($x=h_{1}$, $x=h_{2}$) can be applied to the Equation (1). Then

(3) $$\frac{\tan\;\theta_{1}}{\tan\;\theta_{2}}=\frac{3{h_{1}}^{2}-6dh_{1}+2d^{2}}{3{h_{2}}^{2}-6dh_{2}+2d^{2}}$$

We need to note that the above linearized equation from Bernoulli-Euler equation only works for small angles.

### 2.2. Linear Tapered Model of Whisker

The analytical linear model [19] is based on a cylinder shaped whisker. Here, we derive an equation for a tapered whisker. The linear equation of deflection of a tapered whisker was given by Birdwell et al. [17]. The relationship of bending moment at base, radial distance and tangential angle is

(4) $$E\alpha \frac{dy}{dx}{|}_{x=0}=E\alpha \tan\;\theta_{0}=\left(\frac{L+2d}{6L^{3}}+\frac{1}{d}\left(\frac{d+2L}{6L^{2}}-\frac{1}{3(L-d)}\right)\right)\frac{M}{d}$$

(5) $$E\alpha \frac{dy}{dx}{|}_{x=h}=E\alpha \tan\;\theta_{1}=\left(\frac{L+2d-3h}{6{\left(L-h\right)}^{3}}+\frac{1}{d}\left(\frac{d+2L}{6L^{2}}-\frac{1}{3(L-d)}\right)\right)\frac{M}{d}$$

where $\alpha =\pi /4{\left(r_{base}/4\right)}^{4}$ , *L* is the length of whisker, *d* is the radial distance, *M* is the moment at base and *h* is the sensor position from the base. Dividing Equation (4) by Equation (5), we remove *M* and α. The tangential ratio equation is derived as

(6) $$\frac{\tan\;\theta_{1}}{\tan\;\theta_{0}}=\frac{\left(L+2d-3h\right)/\left[6{\left(L-h\right)}^{3}\right]+\frac{1}{d}\left(\left(d+2L\right)/\left(6L^{2}\right)-1/\left[3\left(L-d\right)\right]\right)}{\left(L+2d\right)/\left(6L^{3}\right)+\frac{1}{d}\left(\left(d+2L\right)/\left(6L^{2}\right)-1/\left[3\left(L-d\right)\right]\right)}$$

(7) $$d=\frac{-B-\sqrt{B^{2}-4AC}}{2A}$$

where *A*, *B* and *C* are the coefficients are defined below:

(8) $$A=\frac{\tan\;\theta_{1}/\tan\;\theta_{0}}{3L^{3}}-\frac{1}{3{\left(L-h\right)}^{3}},\;\;\;\;\;B=\frac{L+3h}{6{\left(L-h\right)}^{3}}-\frac{1}{6L^{2}},\;\;\;\;\;C=\frac{L^{2}-3hL}{6{\left(L-h\right)}^{3}}-\frac{1}{6L}$$

If there are two sensor positions, $h_{1}$ and $h_{2}$, we can still use the tangential ratio to estimate the radial distance (see Appendix A) as follows:

(9) $$\frac{\tan\;\theta_{1}}{\tan\;\theta_{2}}=\frac{\left(L+2d-3h_{1}\right)/\left[6{\left(L-h_{1}\right)}^{3}\right]+\frac{1}{d}\left(\left(d+2L\right)/\left(6L^{2}\right)-1/\left[3\left(L-d\right)\right]\right)}{\left(L+2d-3h_{2}\right)/\left[6{\left(L-h_{2}\right)}^{3}\right]+\frac{1}{d}\left(\left(d+2L\right)/\left(6L^{2}\right)-1/\left[3\left(L-d\right)\right]\right)}$$

The sensor position $h_{2}=0$ leads to Equation (5).

### 2.3. Numerical Method of Whisker Simulation

Previous models are based on the linearized Bernoulli-Euler equation M/EI = dϕ/ds = $-d^{2}y/dx^{2}$. The actual Bernoulli-Euler equation can be written as follows:

(10) $$\frac{1}{r}=\frac{M}{EI}=\frac{d\phi }{ds}=-\frac{d^{2}y/dx^{2}}{{\left[1+{\left(dy/dx\right)}^{2}\right]}^{3/2}}$$

where the bending moment is proportional to the curvature, *r* is the radius of curvature, the tangential angle of the beam is defined as ϕ, and the curvature is given by dϕ/ds where *s* is the arc length along the beam. Since it is difficult to solve Equation (10), a numerical method is used. For this, the whisker can be divided into *N* elements. In order to know how the whisker shaft will deflect, $M_{i}/EI_{i}$ for each element *i* should be given [21].

When a force and its location are given, the moment *M* for each point on the whisker can be known, since $M_{i}=r_{i}\times F$ where $M_{i}$ is the moment on the *i*-th element, $r_{i}$ is the distance between the force and the element, and $I_{i}$ is the moment of inertia of the *i*-th element. After calculating $M_{i}/EI_{i}$ for i=1,...,N, ϕ can be obtained as well by integrating M/EI over ds. Since ϕ is known, (x,y) position for every element can be estimated. Hence, the whole deflection can be reconstructed. However, it should be noted that when (x,y) position for each element changes, so will $r_{i}$ and ultimately $M_{i}/EI_{i}$ will change. Hence, this calculation should be done iteratively until the results converge. The results can be validated by inserting *x* and *y* in $-d^{2}y/dx^{2}/{\left[1+{\left(dy/dx\right)}^{2}\right]}^{3/2}$ in Equation (11) to see if it is identical to $\frac{M}{EI}$.

### 2.4. Passive Sensing

Active whisking needs a rotational movement to sweep a whisker close at the base—see Figure 1. In contrast, a point load (force) is applied to a static cantilever arm with fixed-free condition. This condition is different from active sensing, but the deflection of the beam can be observed. We call it passive sensing. If there are two sensors to measure the tangential angle or slope angle, the tangential ratio can estimate the radial distance as follows:

(11) $$\frac{\tan\;\theta_{1}}{\tan\;\theta_{2}}=\left(\frac{dy}{dx}{|}_{x=h_{1}}\right)/\left(\frac{dy}{dx}{|}_{x=h_{2}}\right)=\frac{{\left(L-h_{2}\right)}^{3}\left[L^{3}\left(L-3h_{1}+2d\right)-{\left(L-h_{1}\right)}^{3}\left(L+2d\right)\right]}{{\left(L-h_{1}\right)}^{3}\left[L^{3}\left(L-3h_{2}+2d\right)-{\left(L-h_{2}\right)}^{3}\left(L+2d\right)\right]}$$

Also, the sensors can measure the deflection displacements at two positions. The ratio of the measurement can produce the distance information.

(12) $$\frac{Y(h_{1})}{Y(h_{2})}=\frac{\left[\frac{d-L}{6{\left(L-h_{1}\right)}^{2}}+\frac{1}{2(L-h_{1})}\right]+C_{1}h_{1}+C_{2}}{\left[\frac{d-L}{6{\left(L-h_{2}\right)}^{2}}+\frac{1}{2(L-h_{2})}\right]+C_{1}h_{2}+C_{2}}$$

where $C_{1}$ and $C_{2}$ are constant parameters determined by boundary conditions and Y(x) is the vertical displacement at a given position *x* (see Appendix B and Appendix C).

## 3. Experimental Results

We simulated the whisker deflection based on the models mentioned above using MATLAB 2011a in a computer Intel Core (TM) i5-2500 CPU (3.30 GHz quad-core). The radial distance estimation based on the linear models will be demonstrated and the estimation errors will be compared. We investigate the noise sensitivity of a cylindrical whisker and a tapered whisker to see if there is any advantage of one or the other type of whisker to estimate the radial distance. We will test if the tangential angles at two sensor positions, that is, the tangential ratio is effective for tactile localization of a whisker.

### 3.1. Results with Linear Cylinder-Type Whisker

**Figure 2:**
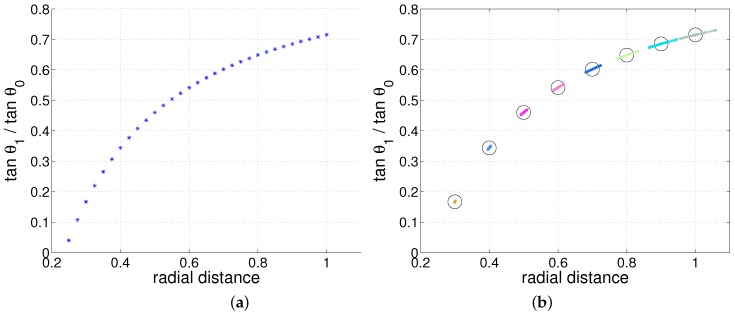
Torque invariance characteristic of analytical model (**a**) x-axis: radial distance, y-axis: $\tan\;\theta_{1}/\tan\;\theta_{0}$, (**b**) The variation of radial distance estimation with noise applied relative to the size of each angle, $\theta_{0}$ and $\theta_{1}$. Using the given analytic equation, radial distance *d* can be estimated with $\tan\;\left(\theta_{1}+\epsilon \right)/\tan\;\left(\theta_{0}+\epsilon \right)$, where ϵ is gaussian noise (sensor position h=0.1).

We initially tested a classical whisker model, a linear cylinder-type whisker. Figure 2a shows the estimation of radial distance depending on the tangential ratio. The robustness to noise using the linear model is shown in Figure 2b. It is shown in Equation (2) that two angle informations are sufficient to estimate the radial distance. We note that when these angle values are noisy, the tangential ratio will vary, giving an estimate with error. Interestingly, Equation (2) shows that without the moment information and Young’s modulus, the radial distance can be estimated.

The moment invariance characteristic in Equation (2) may only be valid because the system is linearized; regardless of any bending moment, the distance can be estimated with the tangential ratio. To see if the moment invariance characteristic holds even for cases where large deflection occurs, the numerical method was used. For the analytical equation, the two parameters to decide the tangential angle $\theta_{1}$ are moment τ and radial distance *d*. In contrast, the numerical method needs a force at the point load *P*, and the point where the contact is held on the whisker.

Therefore, we first take numerical simulation by varying the position of point load and the magnitude of load. From the point load, the whisker deflection can be estimated, and then we can extract the actual radial distance, protraction angle and deflection angle. Hence, we can increase the point load magnitude and change the protraction angle. It should be noted that actual radial distance will generally becomes smaller as the point load magnitude is larger.

**Figure 3:**
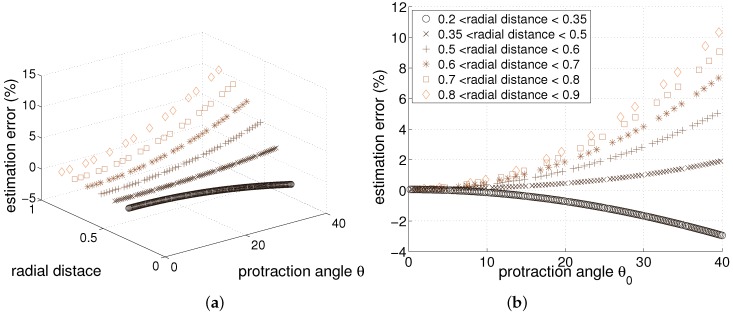
Linear model of cylindrical whisker’s estimation error analysis. The radial distance estimation error (**a**) shown with protraction angle $\theta_{0}$ and radial distance, and (**b**) shown with only protraction angle $\theta_{0}$. The larger the protraction angle, the larger the error becomes as expected (the estimation error is calculated as $\left(d_{actual}-d_{est}\right)/d_{actual}$).

From the simulation results, we can extract the actual protraction angle and deflection angle. Since it is assumed that the whisker sensor will only be given these two values, the estimated radial distance was given by using these two information plugged into the linear model expressed in Equation (2). The estimated radial distance can be compared with the actual radial distance, and the estimation error can be calculated. The radial distance estimation error is defined as

(13) $$err\left(\%\right)=\frac{|d_{actual}-d_{est}|}{d_{actual}}\times 100$$

where $d_{actual}$ denotes actual radial distance and $d_{est}$ denotes estimated radial distance. In Figure 3, the error relationship with protraction angle and the actual radial distance is shown. Because the radial distance cannot be controlled to be constant, the data is discriminated with radial distance ranges.

As expected, when the protraction angle is small, the radial distance estimation error is close to zero. When the protraction angle increases, the magnitude of error will increase. The simulation results also show that when the radial distance is smaller than a certain value, then the radial distance will be overestimated while the other cases will overestimate it. Anyway, the linear model will be quite accurate as long as the protraction angle is under approximately 15 degrees regardless of actual radial distance.

### 3.2. Results with Linear Tapered Model of Whisker

**Figure 4:**
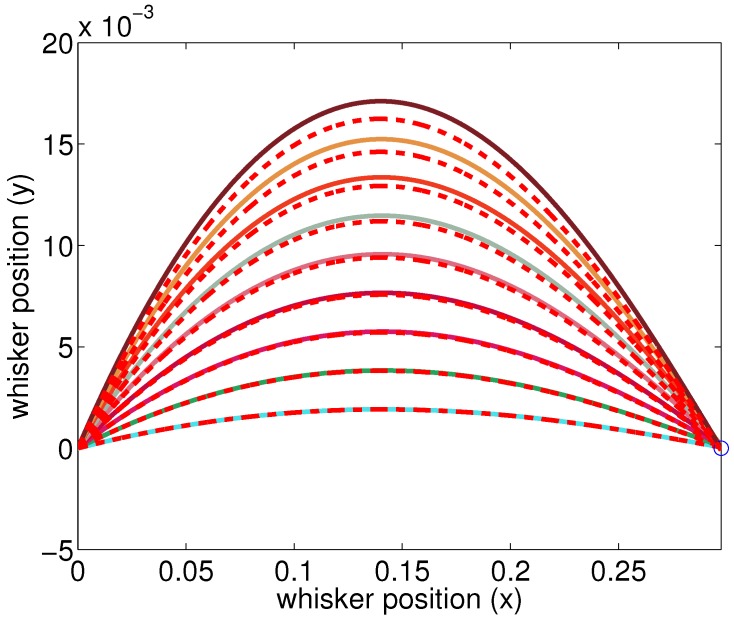
Bending tapered whiskers. Comparison with numerical methods and linear methods. Several cases with different moment values when radial distance is 0.3 are shown. The red dashed lines are the result of linear simulation while the other colored solid lines are the results of the numerical method. It is shown that for small protraction angle, the results are closely matched.

We examined the whisker deflection using linear tapered model. Using the same information, $\theta_{0}$ and $\theta_{1}$, the radial distance can be estimated even with a tapered whisker. To verify if the derived linear equation is correct, simulation results of the linear equation are compared with the numerical method. Such validation is shown in Figure 4. We initially take numerical simulation for a point load with position and magnitude, and estimate the deflection of a tapered whisker. After a numerical simulation, the bending moment at base and the actual radial distance *d* can be extracted. Using these two parameters, the deflection of a tapered whisker based on the linear model can be given. The simulation results are compared and it is clearly seen that when protraction angle is small, the results closely match but the gap increases as the protraction angle increases (or as the point load magnitude increases). This simulation result validates the linear tapered model.

**Figure 5:**
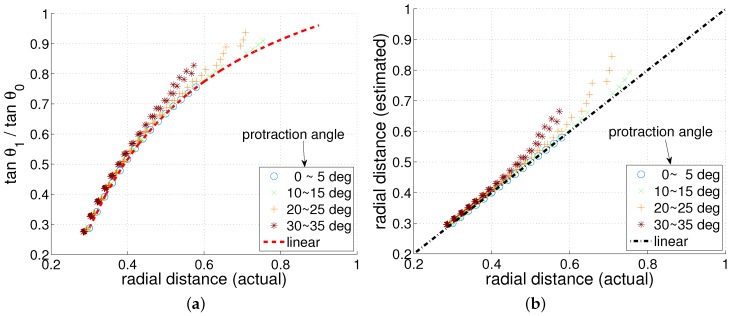
Comparison of results of linear model and numerical method for tapered whisker. (**a**) radial distance (actual) vs. tangential ratio (**b**) radial distance (actual) vs. radial distance (estimated).

Figure 4 gives a rough idea of how the numerical method and the linear model differ for a tapered whisker, and Figure 5 provides a more thorough comparative analysis. In Figure 5a,b, it is clearly seen that the estimated radial distance is always overestimated regardless of protraction angle and radial distance. Also, if the protraction angle and/or radial distance is small, the estimation error will be small as expected.

**Figure 6:**
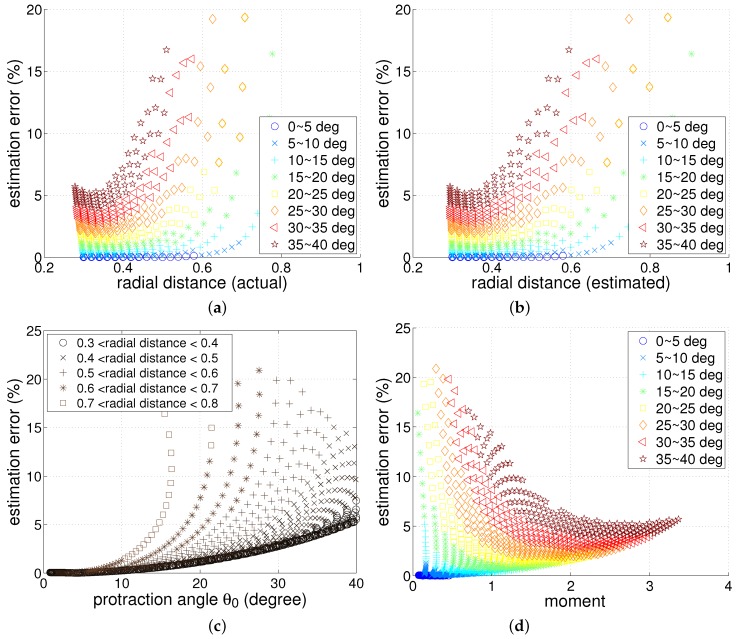
Linear model of tapered whisker’s estimation error analysis (legends in (a), (b), (d) indicate protraction angles); radial distance estimation error shown with (**a**) radial distance (actual) (**b**) radial distance (estimated) (**c**) protraction angle (**d**) bending moment at the whisker base (moment was scaled by assuming E=1 and $r_{base}=1$).

We checked how the parameters such as protraction angle and moment influence the radial distance estimation as shown in Figure 6. The estimation error is involved with those parameters, Figure 6a–d can reveal the main characteristics. Figure 6a,b give similar results since the actual radial distance and estimated radial distance are somewhat similar. From these figures, small angle protraction and small radial distance will result in small estimation error. When protraction angle is large, the estimation error will be large as well. It should be noted that for the tapered whisker case, as the point load is given closer to the tip, the error will greatly increase. This is due to the fact that when the point load is near the tip, the tip will bend significantly since it has a low moment of inertia value which will make large gap between the linear model and numerical method.

Figure 6c shows interesting results. Most of the estimation error for radial distance is closely related to the protraction angle. However, the estimation error will quickly escalate for large radial distances. This result is consistent with Figure 6a,b. Figure 6d shows the relationship between bending moment and estimation error, and the increase of bending moment generally decreases the estimation error. Generally, for the same protraction angle, the larger the radial distance, the smaller the bending moment will be. The high error cases occur where the radial distance becomes close to the whisker length (the contact point close to the tip). For such cases, the moment at base would be small.

If the contact distance is small, large bending moment and small error can be expected. Small protraction angle often has an advantage in maintaining small estimation error. Above all, the radial distance should be smaller than 0.4 times whisker length to keep less than 5% error. That is, if a target object is relatively close to the whisker base, it can reduce the estimation error, although it may need large moment. For an object with small radial distance, a large protraction angle can be allowed to keep the small error. Sample points shown in Figure 6 were collected by changing the point load (force) on many sample positions of the whisker. Subsequently the simulation determines the contact distance and the bending moment as well as the curvature and protraction angle of the whisker. Many samples of varying distances can be observed on the same protraction angle or the same moment as shown in Figure 6.

### 3.3. Noise Robustness Comparison of a Cylindrical and a Tapered Whisker

In the previous sections, the inaccuracy occurrence due to large deflection has been quantified for cylindrical and tapered whisker sensors. For real implementation, it is also very important for whisker sensor systems to be robust to noise. In order to test such robustness for each whisker type, Gaussian noise were added to both protraction angle $\theta_{0}$ and tangential angle $\theta_{1}$ described in Figure 1. Using the two noisy angle information, the radial distance was re-estimated. All the simulations were based on linear models. In order to assume that the simulation results are close to the actual system, the protraction angle for all cases was fixed at 15 degrees.

**Figure 7:**
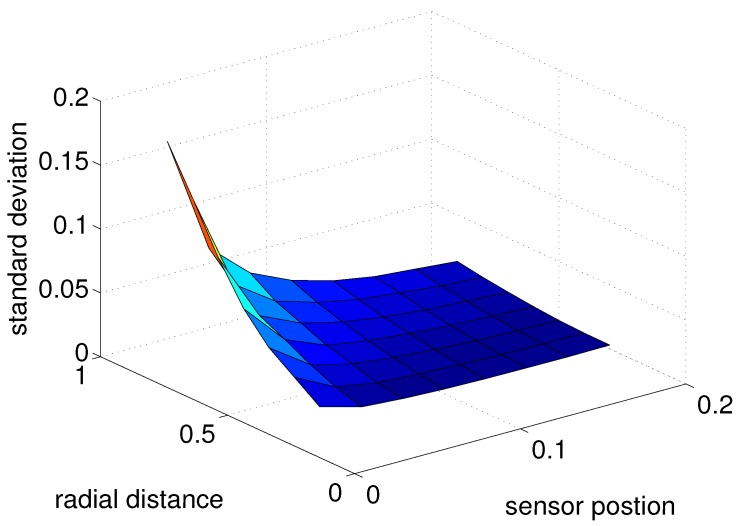
Error standard deviation as a function of radial distance and sensor position with tapered whisker. All the simulations were done when protraction angle $\theta_{0}=15^{\circ }$.

For previous simulations, the sensor position was fixed. However, to see the effect of sensor position as well as the radial distance, both parameters are changed. From Figure 7, it is shown that as the radial distance increases, the estimation error increases for both cases. It can be intuitively understood that the distance of a closer object could be estimated more accurately compared to that of an object at a far distance.

Also, for both cases, the uncertainty or standard deviation will decrease as the sensor position (distance from base) increases. This can be understood intuitively as well since if the sensor is very close to the base, the protraction angle and tangential angle will be almost identical, not giving additional information. However, the sensor position should not be too far or it would become impractical. The difference between the tapered whisker and cylindrical whisker seems to be small in the perspective of standard deviation, but the magnitude of the standard deviation of the tapered whisker sensor is always smaller than of the cylindrical sensor, which means the tapered whisker is more robust to noise when compared to the cylindrical sensor.

**Figure 8:**
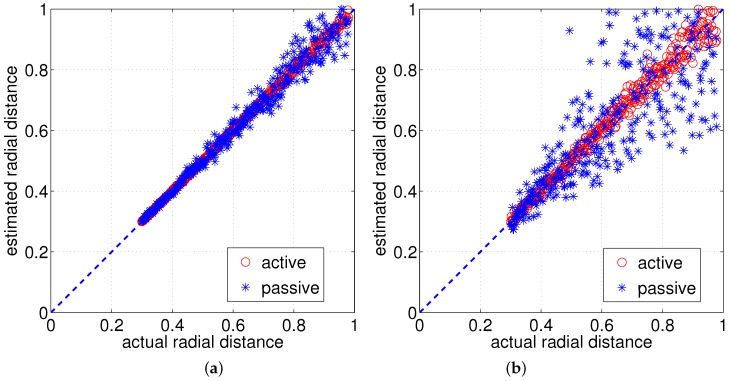
Radial distance estimation using analytical models of active and passive sensing (**a**) tapered case; Gaussian noise [$\sigma =0.1^{\circ }$] applied (**b**) tapered case; Gaussian noise [$\sigma =0.5^{\circ }$].

In Figure 8a,b, different levels of noises were tested. For Figure 8a, Gaussian noise with σ=0.1 degree and for Figure 8b Gaussian noise σ=0.5 degree. While the estimates for small noise, both methods seem to be usable, while the passive sensing method (stationary whisker with object moving towards the whisker sensor) with larger noise seems to be unreliable, unless an appropriate estimation algorithm is employed with multiple measurements.

Here, we derive an analytical model of a tapered whisker. Either passive or active sensing models need at least two different angle information in order to estimate the radial distance without knowing moment, rotational stiffness or force. For the active sensing case, only one deflection angle sensor on the whisker is required if the DC motor has an encoder on it (encoder can measure protraction angle). However, since there is no protraction angle in passive sensing case, at least two deflection angle sensors are required. Putting the two sensor locations at $h_{1}$ and $h_{2}$, and the corresponding tangential angles as $\theta_{1}$ and $\theta_{2}$, the radial distance *d* can be estimated through the following equation.

(14) $$d=\frac{{\left(L-h_{2}\right)}^{3}\left(L^{4}-3h_{1}L^{3}-{\left(L-h_{1}\right)}^{3}L\right)-k{\left(L-h_{1}\right)}^{3}\left(L^{4}-3h_{2}L^{3}-{\left(L-h_{2}\right)}^{3}L\right)}{2k{\left(L-h_{1}\right)}^{3}\left(L^{3}-{\left(L-h_{2}\right)}^{3}\right)-2{\left(L-h_{2}\right)}^{3}\left(L^{3}-{\left(L-h_{1}\right)}^{3}\right)}$$

where $k=\tan\;\theta_{1}/\tan\;\theta_{2}$, the tangential ratio with $\theta_{1}$ and $\theta_{2}$.

**Figure 9:**
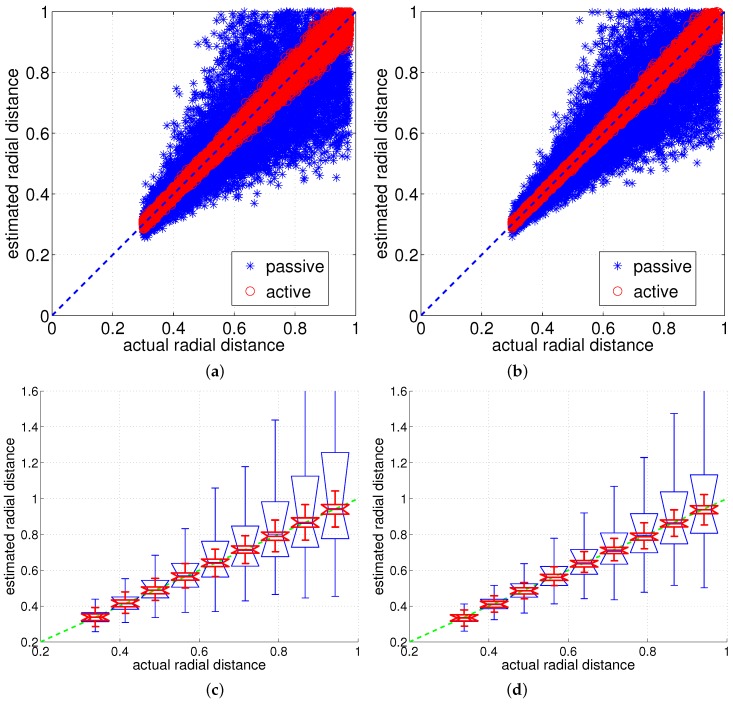
Comparing radial distance estimation using the cylindrical and tapered analytical model for active and passive sensing (distance 1 indicates the tip position) (**a**) cylindrical whisker: Gaussian noise [$\sigma =0.25^{\circ }$] (**b**) tapered whisker: Gaussian noise [$\sigma =0.25^{\circ }$] (**c,d**) the mean and variance of the results of (**a**,**b**).

Figure 9a compares the active and passive sensing using a cylindrical whisker sensor and Figure 9b compares the active and passive sensing using a tapered whisker sensor. Figure 9c,d summarizes the statistical characteristics in distance estimation of the cylindrical and tapered whisker for nine intervals of radial distances by drawing multiple boxplots. Since each boxplot is a summarization of an interval of estimated radial distances, it is bounded to have at least a small value even if the estimated radial distances are exactly the same as the actual radial distances. Nevertheless, the boxplots distinguish the statistical characteristics of distance estimation for active and passive sensing.

From the results of Figure 9, the obvious conclusions are that the active sensing strategy is better than the passive sensing strategy regardless of sensor type (cylindrical or tapered). Also, active tapered whisker is slightly better than the active cylindrical whisker in terms of noise sensitivity, and the passive tapered whisker is better than the passive cylindrical whisker.

### 3.4. Practical Considerations

While all the analytical models and numerical methods for tapered whiskers assumed that the whisker is an ideal cone, either for actual animals and sensor manufacturing, such ideal shape cannot exist. Even if one attempts to make one, the tip will break with great ease. Williams and Kramer [42] measured the taper ratio of eleven types of animal whiskers, three whiskers for each animal and found that some animals have small taper ratio whiskers (4∼9) while other animals, including the rat and the mouse, had large taper ratio (10∼24), where the taper ratio is defined as $ratio=r_{base}/r_{tip}$.

**Figure 10:**
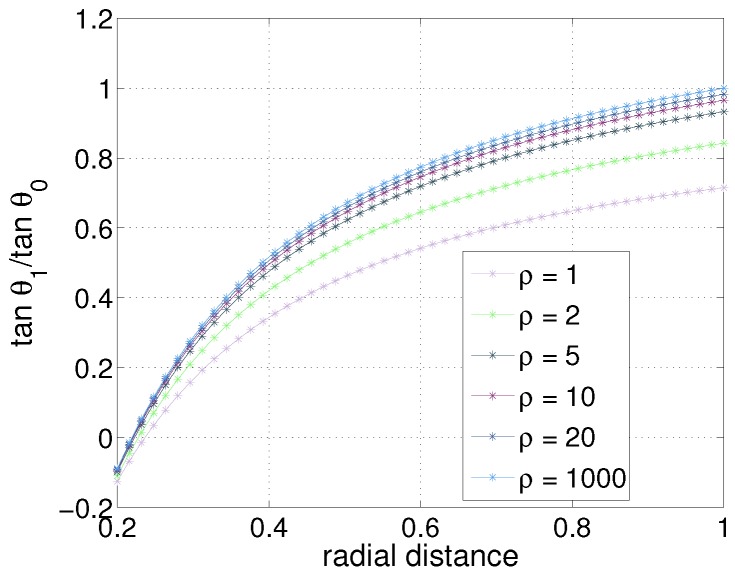
Simulations with different types of taper ratio. Taper ratio, defined as $\rho =r_{base}/r_{tip}$ varies from 1 to 1000. Taper ratio 1 means that it is a cylinder-type whisker, and taper ratio 1000 indicates a high slope of a tapered whisker (tip radius being close to zero).

In Figure 10, the relationship of radial distance - tangential ratio for various cases with different taper ratio is shown. It can be seen that the taper ratio 1 (cylindrical whisker) to 5 differ greatly, 5 to 10 has a relatively small difference, and 10 to 1000 has a smaller difference. Hence, if the taper ratio is approximately around 10, it could be regarded as a very close approximate of a tapered whisker with almost zero tip width. We need to assume that the overall moment of inertia is similar for such approximation.

**Figure 11:**
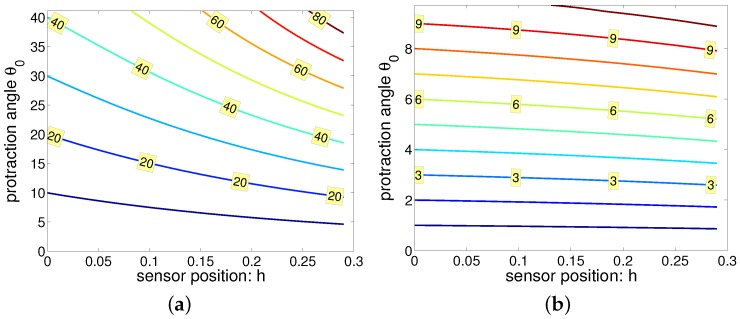
Tangential angle when the protraction angle and sensor position vary. (**a**) radial distance fixed to 0.5, (**b**) radial distance fixed to 0.9. The tangential angle shown in (a) monotonically increases when protraction angle increases. The tangential angle may decrease at a certain point in (b).

In Figure 11, the tangential angle $\theta_{1}$ is given as a function of sensor position *h* and protraction angle $\theta_{0}$. While Figure 11a, where radial distance is 0.5, the protraction angle could be extended to approximately 45 degrees, and Figure 11b had radial distance 0.9 and the protraction angle could be extended to approximately 10 degrees. From the simulation, we saved only the data when the deflected whisker’s tip had the largest distance in the *x*-axis. When the radial distance was large, the tip bended severely making the tip bend inside. Hence, Figure 11b only showed data for protraction angle within 10 degrees.

From Figure 11, two conclusions could be made. First, tapered whiskers touching objects with its tip would flick due to its small rotational stiffness. This result is consistent with the results by Williams and Kramer [42]. Since the difference between protraction angle and tangential angle is very small when the radial distance is large, it would be more difficult to discriminate radial distances. In other words, it is more vulnerable to noise.

### 3.5. Radial Distance Estimation Based on Deflection Displacement

One could estimate the radial distance with two different deflection measurements (vertical deflection). The deflection at a point and the moment can be expressed, assuming the whisker is of cylindrical shape, as

(15) $$EIy\left(x=h_{1}\right)=\frac{-\tau h_{1}\left(h_{1}^{2}-3dh_{1}+2d^{2}\right)}{6d}$$

(16) $$EIy\left(x=h_{2}\right)=\frac{-\tau h_{2}\left(h_{2}^{2}-3dh_{2}+2d^{2}\right)}{6d}$$

where $h_{1}$ and $h_{2}$ are arbitrary points on the whisker, *d* is the radial distance, y(x=h) is the vertical deflection at x=h and τ is the bending moment sensed at base. Using Equations (15) and (16), the radial distance could be estimated from the following equation.

(17) $$\frac{y(x=h_{1})}{y(x=h_{2})}=\frac{h_{1}\left(h_{1}^{2}-3dh_{1}+2d^{2}\right)}{h_{2}\left(h_{2}^{2}-3dh_{2}+2d^{2}\right)}$$

Like the other methods, using deflection displacement could also cancel the moment term. For this method, there should be two sensors along the whisker sensor for radial distance estimation.

## 4. Discussion

There are methods to estimate the radial distance of a cylindrical whisker. In this paper, we introduce two active whisking methods of distance estimation for tapered whiskers. One method uses two different angle information, protraction angle and deflection angle. This method seems similar to the approach using a torque sensor and an angular sensor [20,28], while the method handles the deflection angle. The other model is based on two different deflection measurements. The idea can be applied to both active sensing and passive sensing. The potential or limitation of localization with passive sensing has not been studied in detail. We explored the potential in this paper.

We show an approach which can evaluate the accuracy of linear models in large deflection angles. Through simulations which compare active and passive sensing as well as cylindrical (untapered) and tapered whiskers, it could be concluded that active sensing provides better accuracy and noise-robustness for distance estimation than passive sensing, and a tapered whisker has an accuracy of estimating the radial distance, compared to a cylindrical whisker. The distance estimation is based on deflection angles at two different sensor positions, or the protraction angle and deflection angle.

The results may be consistent with the work of Michenson et al. [43] since the deflection of the whisker is correlated with the bending force in the follicle. From the results, we argue that tapered whiskers have more advantages than cylindrical whiskers both in engineering and biological perspectives.

### 4.1. Tapered Whisker

Tapered whiskers are often found in animals including rodents. Such tapered structure with small cross section area at whisker tip has many advantages; it allows the whisker to probe small surface features, it maintains smaller deflection angle when whisker is passively sensing a moving object, compared to an untapered whisker which cannot be expected to have a large curvature, and also it has robustness in resonant frequency when the tip of the whisker breaks [42].

In our paper, we attempted to observe more advantages of a tapered whisker sensor in an engineering perspective. We derived a new model for tapered whiskers. The model tested by Birdwell et al. [17] estimates the radial distance by measuring the moment or the derivative of moment at base. However, our model uses the protraction angle and a single deflection angle on the whisker shaft without measuring the bending moment. The model shows the radial distance can be predicted without Young’s modulus information or moment related to the material property.

This model is based on a linearized Bernoulli-Euler equation, which means that the accuracy will drop when the assumption of small angle approximation breaks. To see how well this linear equation holds for large protraction angles, numerical solutions were compared with the linear model results. The results showed that even when there is a difference between the linear model and numerical model, the error could be predicted depending on the radial distance, and more accurate estimation could be compensated in this way. To estimate the radial distance, possibly rodents might use an analogy of the protraction angle and deflection angle rather than moment information dependent on the material property of the whisker or the whisker shape.

Our experiments showed that tapered whiskers have better precision of distance estimation in a noisy environment than cylindrial whiskers. The effect can be observed in both active and passive sensing.

### 4.2. Active and Passive Sensing

In the experiments, active and passive sensing for two types of whiskers, cylindrical whiskers and tapered whiskers were compared, respectively. Though the simulations are based on linear models, it would still give insight of active and passive sensing. The results showed that active sensing is more robust to noise for both of the whisker types. We note that tapered whiskers are better than cylindrical whiskers in both active and passive sensing. Also, active sensing is beneficial in radial distance estimation. Th results might provide a hypothesis of why rats actively move their whiskers for tactile perception.

One may argue that rats sense the change of deflection angle rather than the deflection angle itself, similar to the claim of Solomon and Hartmann [20] that rats would sense the change of moment rather than the moment itself. Recently Merkel and slow adapting afferents in the follicle of rats have sensitivity to bending moment and its rate of change [44]. The active tapered whisker model could be differentiated with time to make a model based on the change of tangential angle.

(18) $$\begin{array}{c} \frac{\frac{\partial }{\partial t}\left(\tan\;\theta_{1}\right)}{\frac{\partial }{\partial t}\left(\tan\;\theta_{2}\right)}=\frac{sec^{2}\theta_{1}\cdot \dot{\theta_{1}}}{sec^{2}\theta_{2}\cdot \dot{\theta_{2}}} \end{array}$$

(19) $$\begin{array}{c} \approx \frac{\dot{\theta_{1}}}{\dot{\theta_{2}}} \end{array}$$

From Equation (A38), it is shown that given the protraction angle, the tangential angle and their change, the radial distance could be estimated. It should be noted that the above equation is based on the assumption that the radial distance does not change with time. The detailed derivation is given in the appendix.

For active whisking, measurements of tangential angle at two positions $h_{1}$ and $h_{2}$ can determine the radial distance. However, if they are too close, the tangential ratio becomes close to 1 and the distance estimation can be inaccurate and sensitive to noise. If the two measurements of tangential angles are different, more accurate estimation will be possible. However, if the two positions are too far, the deflection estimation at a position far from the whisker base becomes inaccurate, which may produce relatively large error in distance estimation. Recently it is reported that primary whisker neurons encode whisker curvature, not whisker angle during active sensation [45] and also the Merkel cells and slow adapting cells in the follicle respond to the bending moment and the rate of moment change [44]. If $h_{2}=0$ is applied to the tangential ratio equations, $\theta_{2}$ becomes the protraction angle and it corresponds to the whisker angle. Possibly the tangential angle at another position along the whisker arc could be measured and the tangential ratio could be estimated. There are many mechanosensors inside the follicle, responding to the whisker deflection. The primary afferent neurons at two points along the longitudinal direction of the whisker shaft in the follicle might sense the whisker bending observed at different arc positions of whisker above the skin. Possibly the ratio of the measurements can determine the radial distance.

For passive sensing, deflection measurements at two different positions can estimate the radial distance. In a passive sensing mode, the whisker is influenced by the external force, while active sensing is involved with a torque or bending moment caused by self-generated movements. We assumed the fixed-free condition of a cantilever beam for passive sensing, where the slope at the whisker base is zero. However, the rat whiskers have a pivot point and different analysis may be needed to explain the whisker bending of rat whiskers. Our analysis and results for passive sensing may be quite different from those of biological whiskers.

### 4.3. Re-Tuning for Whisker Length and Thickness Change

If the length and thickness of the whiskers have no change, rats would be able to acquire information from its whiskers in a consistent way. However, there will be cases when short whiskers will grow longer due to whisker breakage or perhaps because the rat whiskers are not fully grown. For either case, the whisker length and possibly the thickness of the whisker will constantly change in the whisker growing process. Hence, if the rats actually use radial distance estimation methods based on either moment or change in moment [20], tangential angle or change in tangential angle, then the rats will need to re-tune its brain due to the characteristics change of the whisker.

Assuming that the growth of rat whisker will change only the length, or length and radius of the base together, the robustness to such change could be analyzed using the deflection equation for tapered whiskers. If the rats use radial distance estimation methods based on torque or change in torque information, the variation will exist for both cases of variation (length only or length and whisker base radius). For this method, if the length changes (but not the base radius), torque term will become more dominant but since the whisker will become more slender, the torque or change of torque at base will fall. Therefore, the re-tuning process would differ for case by case. If both the whisker length and the base of radius change as well, the torque term will become less dominant, but for this case as well, the whisker will become thicker which will make the torque value higher. For this method, all the variables are intertwined making it inconclusive.

If the whisker used tangential angle information, and only the whisker’s length grow, than for that case the tapered whisker will become more like a cylindrical whisker since the previous tip part would become more thicker. If the radius of the base changes as well as the length (in the same ratio), than the normalized results for both cases will be identical. For better analysis, the growth rate of the actual whisker (for the length and base of diameter) would be acquired as well as the actual whisker’s mechanical characteristics for numerical validation of the proposed hypothesis.

### 4.4. Radial Distance Estimation with Only Tangential Angle by Active Whisking

The radial distance method for tapered whiskers used protraction angle and a tangential angle, assuming that the rat will be able to know both information. However, it could be assumed that the rat could sense tangential angles from the follicle, but not the protraction angle. If this is the case, a simple modification of the original tapered whisker model can be shown. Assuming that the follicle can measure at least two tangential angles inside the follicle, the radial distance can be estimated as

(20) $$\frac{\tan\;\theta_{1}}{\tan\;\theta_{2}}=\frac{\frac{L+2d-3h_{1}}{6{\left(L-h_{1}\right)}^{3}}+\frac{1}{d}\left(\frac{d+2L}{6L^{2}}-\frac{1}{3(L-d)}\right)}{\frac{L+2d-3h_{2}}{6{\left(L-h_{2}\right)}^{3}}+\frac{1}{d}\left(\frac{d+2L}{6L^{2}}-\frac{1}{3(L-d)}\right)}$$

This equation implies that the ratio of two tangential angles at two different positions along the longitudinal axis of the follicle can determine the radial distance without any information of elastic property or thickness of whisker.

### 4.5. Future Works

In this paper, we showed simulation results. Therefore, for future works, actual experiments should be conducted in order to validate our results. Also, it could be better if analytical equations could be derived from the nonlinear Bernoulli-Euler equation for results with high precision and fast computation. Nevertheless, the results shown in this paper could be meaningful since it shows the advantage of tapered whiskers in both engineering and biological perspectives.

The actual implement of whisker sensors is another issue. Since there are three different types of radial distance estimation methods, each method could be chosen for user-specific needs. It might be better to use a tapered whisker for more accurate results. There may be a case where deflection measurement is easier than deflection angle. In such case, the third model in this paper could be used.

If one uses a tapered whisker sensor, there is a possibility that the tip will break, leaving the remaining whisker damaged. However, since there may be a curvature change only within the contact point, it is not an important issue whether the tip is broken or not. Even though a tapered whisker sensor with a broken tip will no longer be able to measure the radial distance as far as before, the radial distance estimation method does not change, since the distance estimation only depends on the deflection angles at two different positions, or the protraction angle and deflection angle, not on the material property of the whisker or the moment at base.

Putting all the facts described above in mind, a practical algorithm which could allow the whisker sensor to estimate radial distance in real time with high precision could be tested with biological and artificial whiskers.

Rats normally use multiple whiskers for probing objects. The barrel cortex of rodents processes multiple whiskers in parallel [40,41]. It seems that they collect information obtained from multiple whiskers, and process the knowledge of environmental situation. The distance and texture information can be more accurate if more whiskers probe the same object. For future work, we will see if multiple whiskers can have more advantages of processing tactile signals. The analysis with multiple whiskers may reveal a high level of tactile processing including shape recognition or texture discrimination. In this paper, we showed an analysis of tapered whiskers and the simulation for radial distance estimation. For future work, we can design artificial whiskers to test an advantage of the tapered whiskers. 

## Appendix A. Radial Distance Estimation by Active Sensing with a Tapered Whisker

In this section, the radial distance estimation method using a tapered whisker with two different angle information (protraction angle and tangential angle at an arbitrary point on the whisker) is derived.

First, the linear deflection equation was derived in Birdwell et al. [17]. The following equations summarize the derivation. First of all, the moment or torque on the whisker can be expressed as

(A1) M(x)=F(d−x)for 0≤x≤d

where *d* is the contact distance and *x* is the measured position.

The area moment of inertia of an ideal tapered whisker can be expressed as

(A2) $$I=\frac{\pi r^{4}}{4}$$

(A3) $$r=r_{base}\left(1-\frac{x}{L}\right)$$

(A4) $$I=\frac{\pi }{4}{\left(\frac{r_{base}}{L}\right)}^{4}{\left(L-x\right)}^{4}=\alpha {\left(L-x\right)}^{4}$$

where $\alpha =\frac{\pi }{4}{\left(\frac{r_{base}}{L}\right)}^{4}$.

From the Euler equation,

(A5) $$M\left(x\right)=F\left(d-x\right)=EI\frac{d^{2}y}{dx^{2}}=E\alpha {\left(L-x\right)}^{4}\frac{d^{2}y}{dx^{2}}$$

(A6) $$\frac{d^{2}y}{dx^{2}}=\frac{F}{E\alpha }\frac{d-x}{{\left(L-x\right)}^{4}}$$

(A7) $$\frac{dy}{dx}=\frac{F}{E\alpha }\frac{L+2d-3x}{6{\left(L-x\right)}^{3}}+C_{1}$$

(A8) $$y=\frac{F}{E\alpha }\left[\frac{d-L}{6{\left(L-x\right)}^{2}}+\frac{1}{2(L-x)}\right]+C_{1}x+C_{2}$$

Starting from Equation (A8), Birdwell et al. (2007) showed the analytical solution for a tapered cantilever beam deflection when force and force location are given. While the analytical solution was given in Birdwell et al. (2007) for force on cantilever beam (fixed - free boundary condition), we derived an analytical solution for an active tapered beam. The active whisker or active beam is involved with torque or rotational force as shown in the work [29]. For an active beam, two boundary conditions are given as

(A9) $$\left(1\right)y_{x=0}=0$$

(A10) $$\left(2\right)y_{x=d}=0$$

where *d* is the contact distance.

Using the the first boundary condition (see Equation (A9)) and Equation (A8), we obtain

(A11) $$y\left(x=0\right)=\frac{F}{E\alpha }\left[\frac{d-L}{6L^{2}}+\frac{1}{2L}\right]+C_{2}=0$$

(A12) $$C_{2}=-\frac{F}{E\alpha }\left[\frac{d-L}{6L^{2}}+\frac{1}{2L}\right].$$

where *L* is the whisker length.

The the second boundary condition (Equation (A10)) and Equation (A8) leads to

(A13) $$y\left(x=d\right)=\frac{F}{E\alpha }\left[\frac{d-L}{6{\left(L-d\right)}^{2}}+\frac{1}{2(L-d)}\right]+C_{1}d+C_{2}=0$$

(A14) $$y\left(x=d\right)=\frac{F}{E\alpha }\left[\frac{d-L}{6{\left(L-d\right)}^{2}}+\frac{1}{2(L-d)}\right]+C_{1}d-\frac{F}{E\alpha }\left[\frac{d-L}{6L^{2}}+\frac{1}{2L}\right]=0$$

(A15) $$C_{1}=\frac{F}{dE\alpha }\left(\frac{d-L}{6{\left(L-d\right)}^{2}}-\frac{1}{3(L-d)}\right)=\frac{F}{dE\alpha }\left[-\frac{1}{2(L-d)}\right].$$

Differentiating Equation (A8) with *x*,

(A16) $$\frac{dy}{dx}=\frac{F}{E\alpha }\frac{L+2d-3x}{6{\left(L-x\right)}^{3}}+C_{1}=\frac{F}{E\alpha }\frac{L+2d-3x}{6{\left(L-x\right)}^{3}}+\frac{F}{dE\alpha }\left(\frac{d-L}{6{\left(L-d\right)}^{2}}-\frac{1}{3(L-d)}\right)$$

(A17) $$E\alpha \frac{dy}{dx}=\left[\frac{L+2d-3x}{6{\left(L-x\right)}^{3}}+\frac{1}{d}\left(\frac{d+2L}{6L^{2}}-\frac{1}{3(L-d)}\right)\right]\frac{M}{d}$$

Inserting x=0 and x=h in Equation (A17) can lead to

(A18) $$E\alpha \frac{dy}{dx}{|}_{x=0}=\left[\frac{L+2d}{6L^{3}}+\frac{1}{d}\left(\frac{d+2L}{6L^{2}}-\frac{1}{3(L-d)}\right)\right]\frac{M}{d}$$

(A19) $$E\alpha \frac{dy}{dx}{|}_{x=h}=\left[\frac{L+2d-3h}{6{\left(L-h\right)}^{3}}+\frac{1}{d}\left(\frac{d+2L}{6L^{2}}-\frac{1}{3(L-d)}\right)\right]\frac{M}{d}$$

where x=0 is the whisker base position, x=h is the sensor position to measure the slope and *M* is the bending moment.

Dividing Equation (A19) by Equation (A18),

(A20) $$\frac{{dy/dx|}_{x=h}}{{dy/dx|}_{x=0}}=\frac{\left(\frac{L+2d-3h}{6{\left(L-h\right)}^{3}}+\frac{1}{d}\left(\frac{d+2L}{6L^{2}}-\frac{1}{3(L-d)}\right)\right)}{\left(\frac{L+2d}{6L^{3}}+\frac{1}{d}\left(\frac{d+2L}{6L^{2}}-\frac{1}{3(L-d)}\right)\right)}$$

Since dy/dx can be substituted with tanθ for small angle, Equation (A20) can be represented as

(A21) $$\frac{\tan\;\theta_{1}}{\tan\;\theta_{0}}=\frac{\left(\frac{L+2d-3h}{6{\left(L-h\right)}^{3}}+\frac{1}{d}\left(\frac{d+2L}{6L^{2}}-\frac{1}{3(L-d)}\right)\right)}{\left(\frac{L+2d}{6L^{3}}+\frac{1}{d}\left(\frac{d+2L}{6L^{2}}-\frac{1}{3(L-d)}\right)\right)}$$

where $\tan\;\theta_{1}{=dy/dx|}_{1}$ and $\tan\;\theta_{0}{=dy/dx|}_{0}$. The equation can be re-written into a quadratic equation for parameter *d*. We can solve the solution for radial distance *d* while the whisker length *L* is fixed.

The distance estimation only depends on the protraction angle and deflection angle, not on the material property of the whisker or the moment at base. We also note that the area moment of inertia related with the thickness is not a cruicial factor when we assume that the tip width is close to zero.

Using Equation (A21), we can find the radial distance *d* as follows:

(A22) $$d=\frac{-B-\sqrt{B^{2}-4AC}}{2A}$$

where *A*, *B* and *C* are the coefficients defined as follows:

(A23) $$A=\frac{\tan\;\theta_{1}/\tan\;\theta_{0}}{3L^{3}}-\frac{1}{3{\left(L-h\right)}^{3}},\;\;\;B=\frac{L+3h}{6{\left(L-h\right)}^{3}}-\frac{1}{6L^{2}}\;\;\;C=\frac{L^{2}-3hL}{6{\left(L-h\right)}^{3}}-\frac{1}{6L}$$

If there are two sensor positions, $h_{1}$ and $h_{2}$, not the whisker base, to measure deflection of the whisker, the tangential ratio is given by

(A24) $$\frac{\tan\;\theta_{1}}{\tan\;\theta_{2}}=\frac{\frac{L+2d-3h_{1}}{6{\left(L-h_{1}\right)}^{3}}+\frac{1}{d}\left(\frac{d+2L}{6L^{2}}-\frac{1}{3(L-d)}\right)}{\frac{L+2d-3h_{2}}{6{\left(L-h_{2}\right)}^{3}}+\frac{1}{d}\left(\frac{d+2L}{6L^{2}}-\frac{1}{3(L-d)}\right)}$$

## Appendix B. Passive Sensing with a Tapered Whisker

In this paper, passive sensing simulation for tapered whisker was used to compare it with active sensing simulation. The analytic model for passive sensing is derived in this section. Starting from Equation (A8),

(A25) $$y=\frac{F}{E\alpha }\left[\frac{d-L}{6{\left(L-x\right)}^{2}}+\frac{1}{2(L-x)}\right]+C_{1}x+C_{2}$$

and using the boundary conditions $\frac{dy}{dx}{|}_{x=0}$ and ${y|}_{x=0}=0$, the constant can be found. $C_{1}=-\frac{F}{E\alpha }\left[\frac{L+2d}{6L^{3}}\right]$ and $C_{2}=-\frac{F}{E\alpha }\left[\frac{2L+d}{6L^{2}}\right]$. Finally, the equation can be written as

(A26) $$\frac{dy}{dx}=\frac{F}{E\alpha }\left[\frac{L-3x+2d}{6{\left(L-x\right)}^{3}}-\frac{L+2d}{6L^{3}}\right]$$

Since the protraction angle will always be zero, we need two tangential angles measured from the whisker shaft. Naming the position for each sensor as $x=h_{1}$ and $x=h_{2}$ and inserting them into Equation (A26),

(A27) $$\frac{dy}{dx}{|}_{x=h_{1}}=\frac{F}{E\alpha }\left[\frac{L-3h_{1}+2d}{6{\left(L-h_{1}\right)}^{3}}-\frac{L+2d}{6L^{3}}\right]$$

(A28) $$\frac{dy}{dx}{|}_{x=h_{2}}=\frac{F}{E\alpha }\left[\frac{L-3h_{2}+2d}{6{\left(L-h_{2}\right)}^{3}}-\frac{L+2d}{6L^{3}}\right]$$

(A29) $$\frac{\tan\;\theta_{1}}{\tan\;\theta_{2}}=\left(\frac{dy}{dx}{|}_{x=h_{1}}\right)/\left(\frac{dy}{dx}{|}_{x=h_{2}}\right)=\frac{{\left(L-h_{2}\right)}^{3}\left[L^{3}\left(L-3h_{1}+2d\right)-{\left(L-h_{1}\right)}^{3}\left(L+2d\right)\right]}{{\left(L-h_{1}\right)}^{3}\left[L^{3}\left(L-3h_{2}+2d\right)-{\left(L-h_{2}\right)}^{3}\left(L+2d\right)\right]}$$

where we set $k=\left(\frac{dy}{dx}{|}_{x=h_{1}}\right)/\left(\frac{dy}{dx}{|}_{x=h_{2}}\right)=\tan\;\theta_{1}/\tan\;\theta_{2}$.

The final equation for radial distance estimation for passive sensing can be expressed as

(A30) $$d=\frac{{\left(L-h_{2}\right)}^{3}\left(L^{4}-3h_{1}L^{3}-{\left(L-h_{1}\right)}^{3}L\right)-k{\left(L-h_{1}\right)}^{3}\left(L^{4}-3h_{2}L^{3}-{\left(L-h_{2}\right)}^{3}L\right)}{2k{\left(L-h_{1}\right)}^{3}\left(L^{3}-{\left(L-h_{2}\right)}^{3}\right)-2{\left(L-h_{2}\right)}^{3}\left(L^{3}-{\left(L-h_{1}\right)}^{3}\right)}$$

## Appendix C. Radial Distance Estimation Based on Two Deflection Measurements by Passive Sensing

The derivation of the equation of radial distance estimation starts from the

(A31) $$EIy=-\frac{1}{6}x^{3}Fu\left(x\right)+\frac{1}{6}{\left(x-d\right)}^{3}Fu\left(x-d\right)+\frac{1}{6}C_{1}x^{3}+\frac{1}{2}C_{2}x^{2}+C_{3}x+C_{4}$$

where F=M/d. Inserting all the constants yields

(A32) $$EIy=-\frac{1}{6}x^{3}\frac{M}{d}+\frac{1}{2}Mx^{2}-\frac{1}{3}Mdx=\frac{-Mx(x^{2}-3dx+2d^{2})}{6d}$$

Hence, by putting $h_{1}$ and $h_{2}$ in the above equation where $h_{1}≢h_{2}$

(A33) $$EIy\left(x=h_{1}\right)=\frac{-Mh_{1}\left(h_{1}^{2}-3dh_{1}+2d^{2}\right)}{6d}$$

(A34) $$EIy\left(x=h_{2}\right)=\frac{-Mh_{2}\left(h_{2}^{2}-3dh_{2}+2d^{2}\right)}{6d}$$

Using the above two equations, a simple form without momentum can be obtained as

(A35) $$\frac{y(x=h_{1})}{y(x=h_{2})}=\frac{h_{1}\left(h_{1}^{2}-3dh_{1}+2d^{2}\right)}{h_{2}\left(h_{2}^{2}-3dh_{2}+2d^{2}\right)}$$

From the above equation, the radial distance can be estimated with two different deflection measurements. From Equation (A25), the deflection for a tapered whisker is given as

(A36) $$Y\left(x\right)=\frac{F}{E\alpha }\left[\frac{d-L}{6{\left(L-x\right)}^{2}}+\frac{1}{2(L-x)}\right]+C_{1}x+C_{2}.$$

The ratio of $Y\left(h_{1}\right)/Y\left(h_{2}\right)$ can be used to estimate the contact distance.

(A37) $$\frac{Y(h_{1})}{Y(h_{2})}=\frac{\left[\frac{d-L}{6{\left(L-h_{1}\right)}^{2}}+\frac{1}{2(L-h_{1})}\right]+C_{1}h_{1}+C_{2}}{\left[\frac{d-L}{6{\left(L-h_{2}\right)}^{2}}+\frac{1}{2(L-h_{2})}\right]+C_{1}h_{2}+C_{2}}$$

## Appendix D. Radial Distance Estimation from Angular Velocity by Active Whisking

(A38) $$\begin{array}{cc} \frac{\frac{\partial }{\partial t}\left(\tan\;\theta_{1}\right)}{\frac{\partial }{\partial t}\left(\tan\;\theta_{2}\right)} & =\frac{\left(\frac{L+2d-3h_{1}}{6{\left(L-h_{1}\right)}^{3}}+\frac{1}{d}\left(\frac{d+2L}{6L^{2}}-\frac{1}{3(L-d)}\right)\right)}{\left(\frac{L+2d-3h_{2}}{6{\left(L-h_{2}\right)}^{3}}+\frac{1}{d}\left(\frac{d+2L}{6L^{2}}-\frac{1}{3(L-d)}\right)\right)}\cdot \frac{\frac{\partial }{\partial t}\tau \cdot \frac{1}{d}}{\frac{\partial }{\partial t}\tau \cdot \frac{1}{d}}\\ & =\frac{sec^{2}\theta_{1}\cdot \dot{\theta_{1}}}{sec^{2}\theta_{2}\cdot \dot{\theta_{2}}}\\ & \approx \frac{\dot{\theta_{1}}}{\dot{\theta_{2}}} \end{array}$$

## References

[1] Obrist M.K. (1995). Flexible bat echolocation: The influence of individual, habitat and conspecifics on sonar signal design. Behav. Ecol. Sociobiol..

[2] Nelson M.E., Maciver M.A. (1999). Prey capture in the weakly electric fish Apteronotus albifrons: Sensory acquisition strategies and electrosensory consequences. J. Exp. Biol..

[3] Brownell P.H., van Hemmen J.L. How the sand scorpion locates its prey. APS March 2000 Meeting.

[4] Polley D.B., Rickert J.L., Frostig R.D. (2005). Whisker-based discrimination of object orientation determined with a rapid training paradigm. Neurobiol. Learn. Mem..

[5] Dehnhardt G., Mauck B., Hanke W., Bleckmann H. (2001). Hydrodynamic trail-following in harbor seals (Phoca vitulina). Science.

[6] Schulte-Pelkum N., Wieskotten S., Hanke W., Dehnhardt G., Mauck B. (2007). Tracking of biogenic hydrodynamic trails in harbour seals (Phoca vitulina). J. Exp. Biol..

[7] Wieskotten S., Dehnhardt G., Mauck B., Miersch L., Hanke W. (2010). Hydrodynamic determination of the moving direction of an artificial fin by a harbour seal (Phoca vitulina). J. Exp. Biol..

[8] Towal R., Hartmann M. (2006). Right–left asymmetries in the whisking behavior of rats anticipate head movements. J. Neurosci..

[9] Towal R., Hartmann M. (2008). Variability in velocity profiles during free-air whisking behavior of unrestrained rats. J. Neurophysiol..

[10] Szwed M., Bagdasarian K., Ahissar E. (2003). Encoding of vibrissal active touch. Neuron.

[11] Hartmann M. (2009). Active touch, exploratory movements, and sensory prediction. Integr. Comp. Biol..

[12] Lepora N., Evans M., Fox C., Diamond M., Gurney K., Prescott T. Naive Bayes texture classification applied to whisker data from a moving robot. Proceedings of the 2010 International Joint Conference on Neural Networks (IJCNN).

[13] Lepora N., Fox C., Evans M., Mitchinson B., Motiwala A., Sullivan J., Pearson M., Welsby J., Pipe T., Gurney K., Prescott T. A general classifier of whisker data using stationary naive bayes: Application to BIOTACT robots. Proceedings of the 12th Annual Conference of Towards Autonomous Robotic Systems.

[14] Solomon J., Hartmann M. (2008). Artificial whiskers suitable for array implementation: Accounting for lateral slip and surface friction. IEEE Trans. Robot..

[15] Clements T., Rahn C. (2006). Three-dimensional contact imaging with an actuated whisker. IEEE Trans. Robot..

[16] Kaneko M. Active antenna. Proceedings of the IEEE International Conference on Robotics and Automation.

[17] Birdwell J., Solomon J., Thajchayapong M., Taylor M., Cheely M., Towal R., Conradt J., Hartmann M. (2007). Biomechanical models for radial distance determination by the rat vibrissal system. J. Neurophysiol..

[18] Solomon J., Hartmann M. (2011). Radial distance determination in the rat vibrissal system and the effects of Weber’s law. Philos. Trans. R. Soc. B Biol. Sci..

[19] Kim D., Möller R. (2007). Biomimetic whiskers for shape recognition. Robot. Auton. Syst..

[20] Solomon J., Hartmann M. (2006). Biomechanics: Robotic whiskers used to sense features. Nature.

[21] Solomon J., Hartmann M. (2010). Extracting object contours with the sweep of a robotic whisker using torque information. Int. J. Robot. Res..

[22] Schultz A., Solomon J., Peshkin M., Hartmann M. Multifunctional whisker arrays for distance detection, terrain mapping, and object feature extraction. Proceedings of the IEEE International Conference on Robotics and Automation.

[23] Fend M. (2005). Whisker-based texture discrimination on a mobile robot. Lecture Notes in Computer Science.

[24] Kim D., Moeller R. (2014). A biomimetic whisker for texture discrimination and distance estimation. From Animals to Animats 8, Proceedings of the International Conference on Simulation and Adaptive Behavior.

[25] Scholz G., Rahn C. (2004). Profile sensing with an actuated whisker. IEEE Trans. Robot. Autom..

[26] Ueno N., Kaneko M. Dynamic active antenna-a principle of dynamic sensing. Proceedings of the IEEE International Conference on Robotics and Automation.

[27] Kaneko M., Kanayama N., Tsuji T. Vision based active antenna. Proceedings of the IEEE International Conference on Robotics and Automation.

[28] Kaneko M., Kanayama N., Tsuji T. (1998). Active antenna for contact sensing. IEEE Trans. Robot. Autom..

[29] Kim D., Möller R. Passive sensing and active sensing of a biomimetic whisker. Proceedings of the International Conference on the Simulation and Synthesis of Living Systems.

[30] Kottapalli A., Asadnia M., Hans H., Miao J.M., Triantafyllou M. Harbor seal whisker inspired flow sensors to reduce vortex-induced vibrations. Proceedings of the 28th IEEE International Conference on Micro Electro Mechanical Systems.

[31] Kottapalli A., Asadnia M., Hans H., Miao J.M., Triantafyllou M. Harbor seal inspired MEMS artificial micro-whisker sensor. Proceedings of the 27th IEEE International Conference on Micro Electro Mechanical Systems.

[32] Mitchinson B., Prescott T. (2013). Whisker Movements Reveal Spatial Attention: A Unified Computational Model of Active Sensing Control in the Rat. PLoS Comput. Biol..

[33] Rooney T., Pearson M., Welsby J., Horsfield I., Sewell R., Dogramadzi S. Artificial active whiskers for guiding underwater autonomous walking robots. Proceedings of the 14th international Conference on climbing and walking robot and the support technologies for mobile machines.

[34] Towal R., Quist B., Gopal V., Solomon J., Hartmann M. (2011). The morphology of the rat vibrissal array: A model for quantifying spatiotemporal patterns of whisker-object contact. PLoS Comput. Biol..

[35] Hartmann M., Johnson N., Towal R., Assad C. (2003). Mechanical characteristics of rat vibrissae: Resonant frequencies and damping in isolated whiskers and in the awake behaving animal. J. Neurosci..

[36] Quist B., Faruqi R., Hartmann M. (2011). Variation in Young’s modulus along the length of a rat vibrissa. J. Biomech..

[37] Arabzadeh E., Petersen R., Diamond M. (2003). Encoding of whisker vibration by rat barrel cortex neurons: implications for texture discrimination. J. Neurosci..

[38] Arabzadeh E., Zorzin E., Diamond M. (2005). Neuronal encoding of texture in the whisker sensory pathway. PLoS Biol..

[39] Von Heimendahl M., Itskov P., Arabzadeh E., Diamond M. (2007). Neuronal activity in rat barrel cortex underlying texture discrimination. PLoS Biol..

[40] Petersen C. (2007). The Functional Organization of the Barrel Cortex. Neuron.

[41] Feldmeyer D., Brecht M., Helmchen F., Petersen C., Poulet J., Staiger J., Luhmann H., Schwarz C. (2013). Barrel cortex function. Prog. Neurobiol..

[42] Williams C., Kramer E. (2010). The advantages of a tapered whisker. PLoS ONE.

[43] Mitchinson B., Gurney K., Redgrave P., Melhuish C., Pipe A., Pearson M., Gilhespy I., Prescott T. (2004). Empirically inspired simulated electro-mechanical model of the rat mystacial follicle-sinus complex. Proc. R. Soc. Lond. Ser. B Biol. Sci..

[44] Severson K., Xu D., de Loo M.V., Bai L., Ginty D., O’Connor D. (2017). Active Touch and Self-Motion Encoding by Merkel Cell-Associated Afferents. Neuron.

[45] Campagner D., Evans M., Bale M., Erskine A., Petersen R. (2016). Prediction of primary somatosensory neuron activity during active tactile exploration. Elife.

